# Supplementary figures and images for: AMPK regulates ESCRT-dependent microautophagy of proteasomes concomitant with proteasome storage granule assembly during glucose starvation
Source: PLoS Genet. 2019 Nov 18;15(11):e1008387. doi: 10.1371/journal.pgen.1008387 (PMC6886873; doi:10.1371/journal.pgen.1008387)

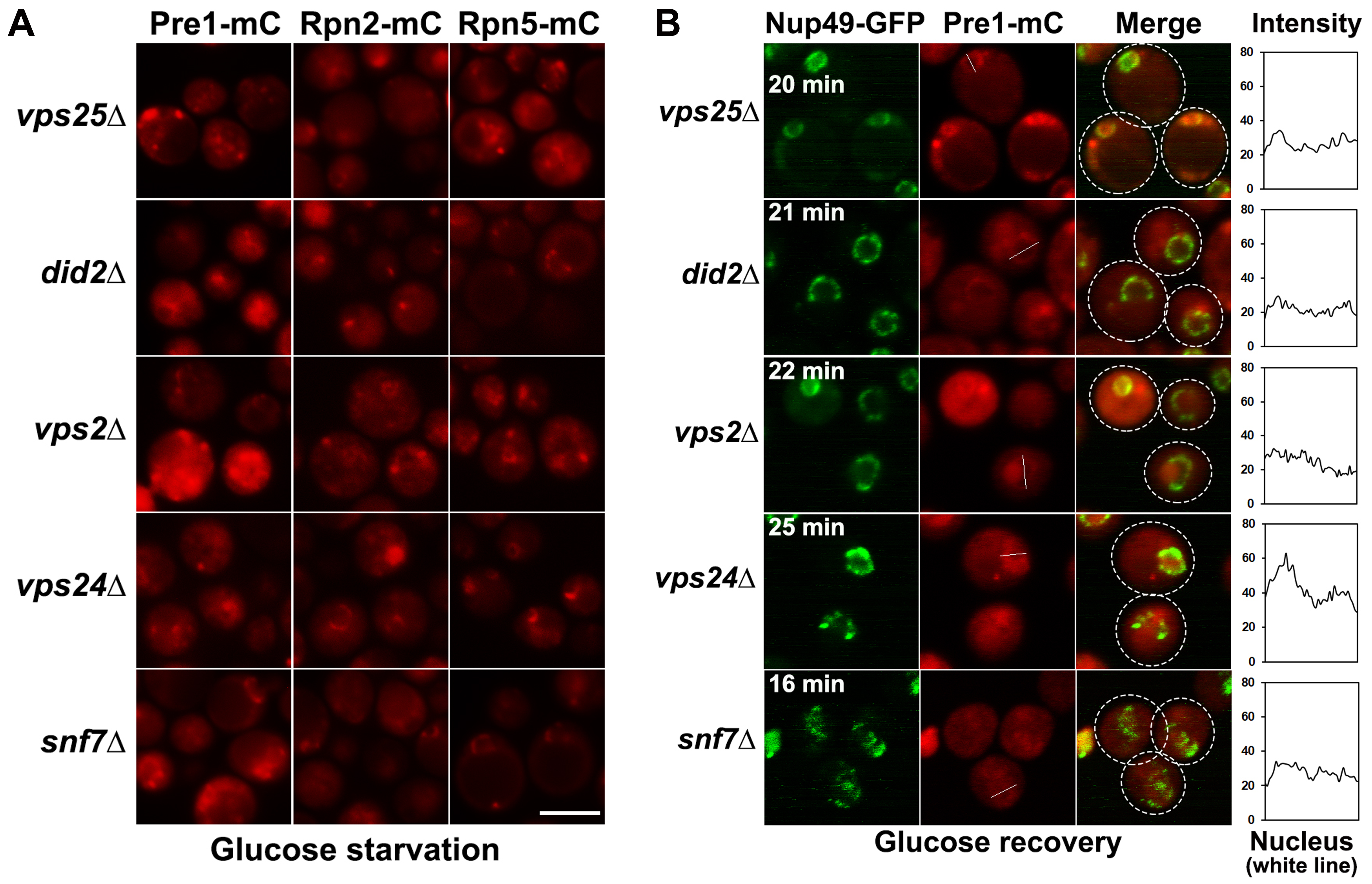

Supplement: S1 Fig — (A) Epifluorescence images of Pre1-mC, Rpn2-mC, and Rpn5-mC in ESCRT-defective mutant cells (ESCRT-II: vps25Δ, and ESCRT-III: did2Δ, vps2Δ, vps24Δ, snf7Δ) during low glucose starvation. Scale bar, 5 μm. (B) Confocal images of Pre1-mC in the ESCRT mutant cells after glucose recovery at the indicated time points. The times indicate when the images were taken after glucose add-back; Nup49-GFP served as a nuclear envelope marker; white dotted circles indicate the approximate outlines of cells. The line charts quantify signal intensity of Pre1-mC in the indicated white line across the nucleus after glucose recovery. (TIF) [file pgen.1008387.s001.tif]

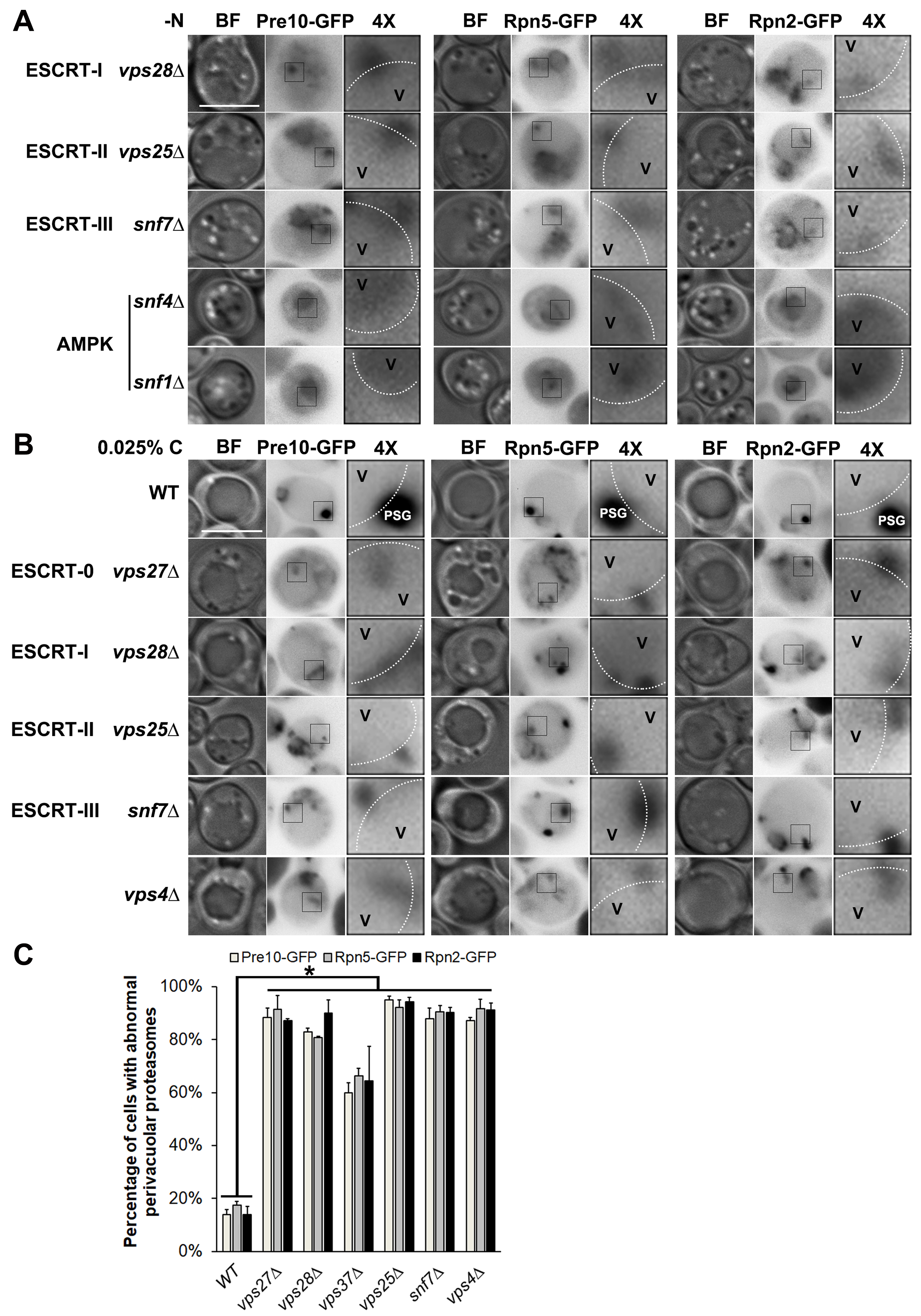

Supplement: S2 Fig — (A) Epifluorescence images of Pre10-GFP, Rpn5-GFP, and Rpn2-GFP in nitrogen-starved ESCRT mutant (vps28Δ, vps25Δ, and snf7Δ) and AMPK mutant (snf4Δ, snf1Δ) cells from figure panel (2A). (B) Epifluorescence images of Pre10-GFP, Rpn5-GFP, and Rpn2-GFP in low glucose-starved WT and ESCRT mutant cells from figure panel (3A). The vacuolar membrane is marked with dotted white line. BF: bright field. V: vacuole. 4×: 4× enlargement of the squared regions in cells. Scale bars, 5 μm. (C) Quantification of cells with abnormal perivacuolar proteasomes in WT and mutant cells in low glucose used in panel (B). Cells counted (Pre10-GFP, Rpn5-GFP, Rpn2-GFP): WT (278, 273, 391), vps27Δ (274, 265, 434), vps28Δ (437, 386, 253), vps37Δ (359, 323, 426), vps25Δ (312, 246, 319), snf7Δ (233, 253, 325), and vps4Δ (301, 336, 437). Results plotted as mean±sd. *, P<0.05 (ANOVA single factor analysis comparing ESCRT mutants to WT). (TIF) [file pgen.1008387.s002.tif]

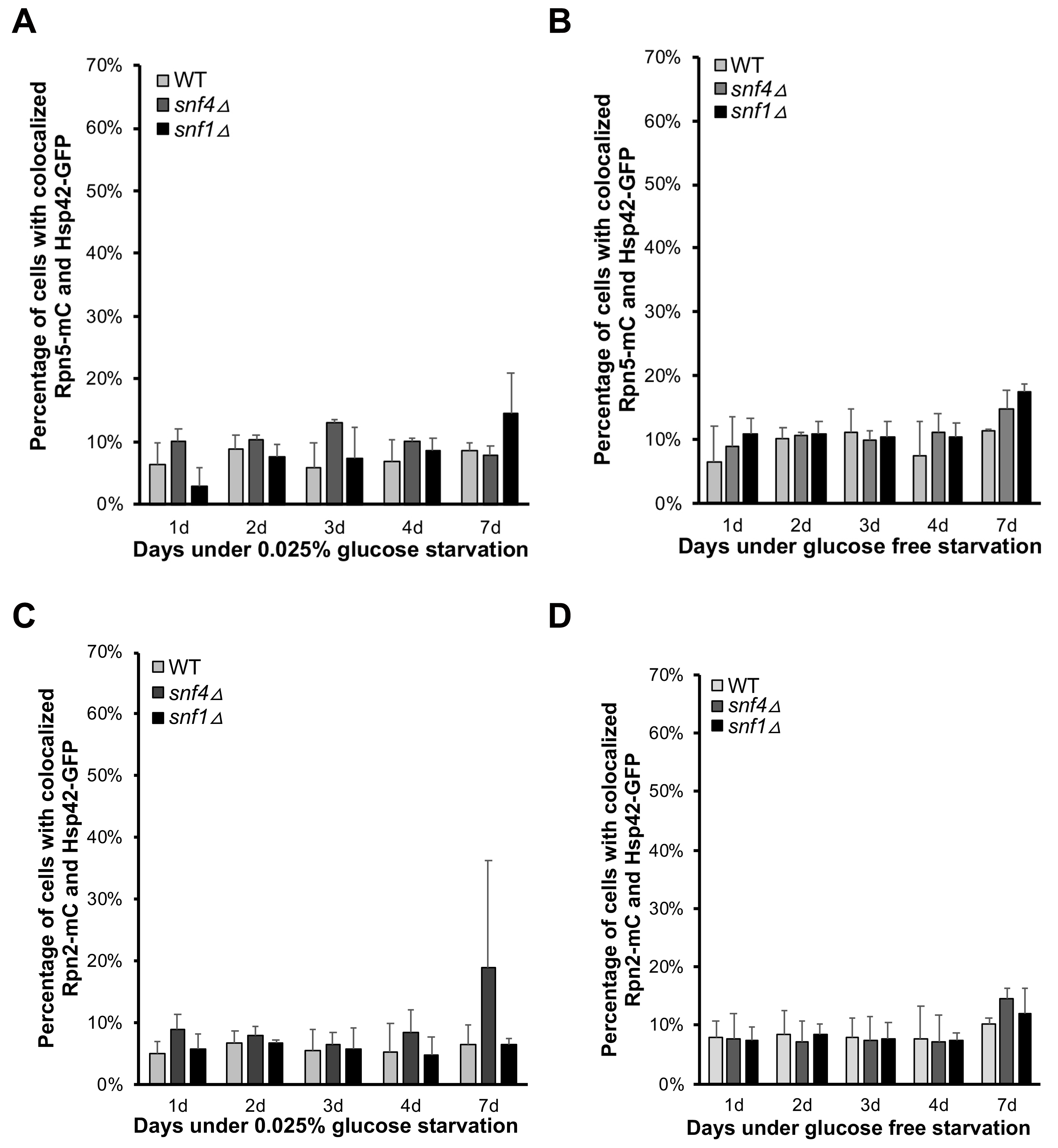

Supplement: S3 Fig — (A) Quantification of colocalized Rpn5-mC and Hsp42-GFP in WT (577 cells counted [1d], 542 [2d], 748 [3d], 792 [4d], 446 [7d]), snf4Δ (546 [1d], 790 [2d], 838 [3d], 368 [4d], 524 [7d]), and snf1Δ (316 [1d], 656 [2d], 530 [3d], 374 [4d], 266 [7d]) live cells in 0.025% glucose. (B) Percentage of living cells with colocalized Rpn5-mC and Hsp42-GFP. WT (292 cells counted [1d], 580 [2d], 457 [3d], 419 [4d], 301 [7d]), snf4Δ (622 [1d], 547 [2d], 403 [3d], 440 [4d], 164 [7d]), and snf1Δ (555 [1d], 465 [2d], 422 [3d], 434 [4d], 212 [7d]) cultures were grown in glucose-free medium. (C) Quantification of colocalized Rpn2-mC and Hsp42-GFP in WT (469 cells counted [1d], 402 [2d], 498 [3d], 460 [4d], 435 [7d]), snf4Δ (355 [1d], 573 [2d], 704 [3d], 494 [4d], 499 [7d]), and snf1Δ (459 [1d], 445 [2d], 555 [3d], 535 [4d], 348 [7d]) live cells in 0.025% glucose. (D) Percentage of living cells with colocalized Rpn2-mC and Hsp42-GFP. WT (347 cells counted [1d], 481 [2d], 601 [3d], 396 [4d], 367 [7d]), snf4Δ (471 [1d], 541 [2d], 352 [3d], 385 [4d], 183 [7d]), and snf1Δ (563 [1d], 415 [2d], 325 [3d], 347 [4d], 138 [7d]) cultures were grown in glucose-free medium. Results plotted as mean±sd.. (TIF) [file pgen.1008387.s003.tif]

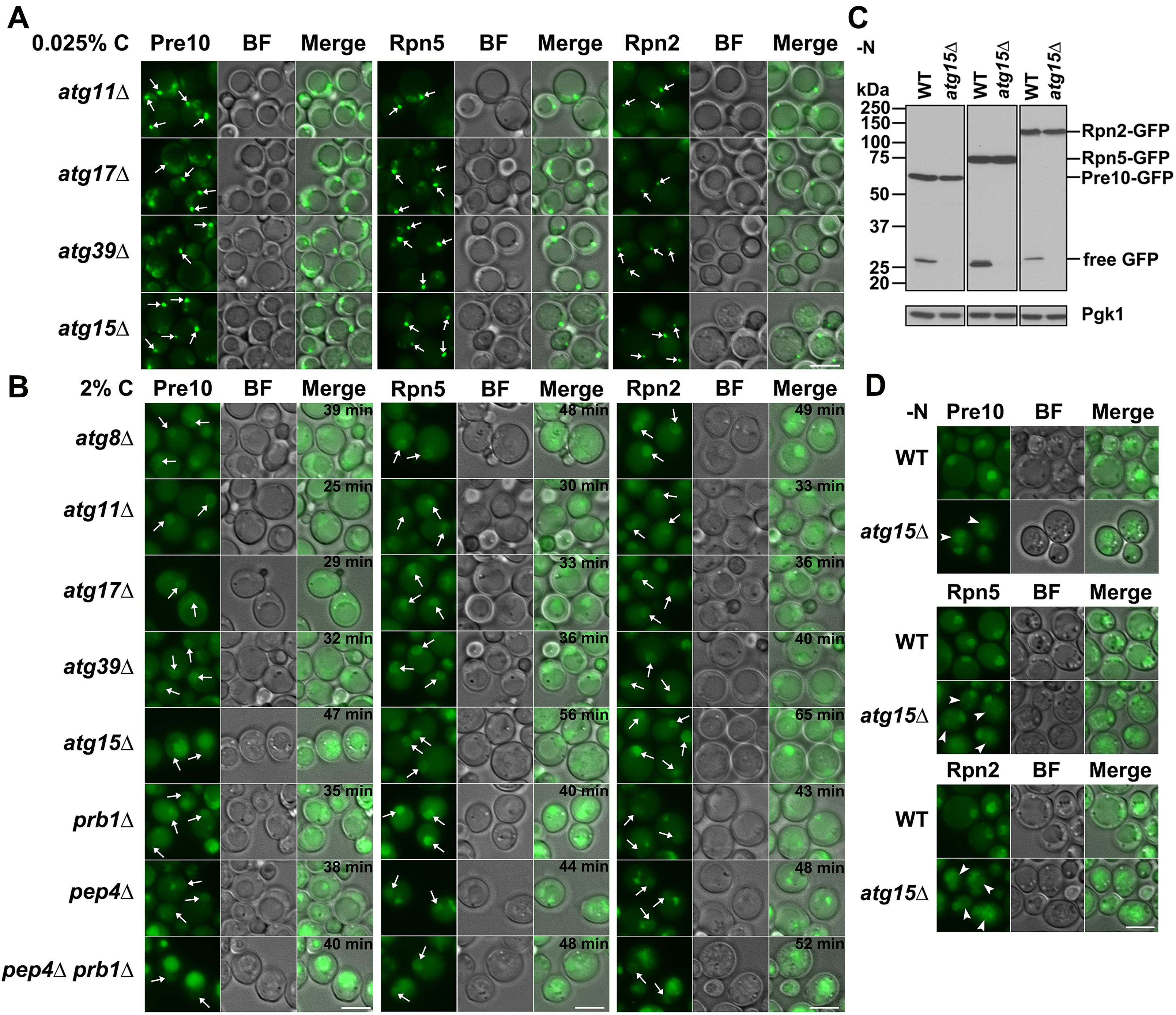

Supplement: S4 Fig — (A) Epifluorescence images of Pre10-GFP, Rpn5-GFP, and Rpn2-GFP in low glucose-starved core macroautophagy mutants (atg11Δ, atg17Δ, atg39Δ, atg15Δ) from figure panel (5A). (B) Epifluorescence images of core macroautophagy mutants (atg8Δ, atg11Δ, atg17Δ, atg39Δ, atg15Δ) and vacuolar protease-deficient mutants (prb1Δ, pep4Δ, pep4Δ prb1Δ) cells at the indicated time recovery in 2% glucose. Cells were from figure panel (5A). White arrows mark PSGs in panel (A) and the nucleus in panel (B). (C) Anti-GFP immunoblot analyses of WT and a vacuolar lipase-deficient mutant atg15Δ cells under nitrogen starvation for ~1 day at 30°C. (D) Epifluorescence images of nitrogen-starved WT and atg15Δ cells from panel (C). White arrowheads mark GFP-tagged full length proteasomes in the vacuole lumen in atg15Δ cells. BF: bright field. Scale bars, 5 μm. (TIF) [file pgen.1008387.s004.tif]

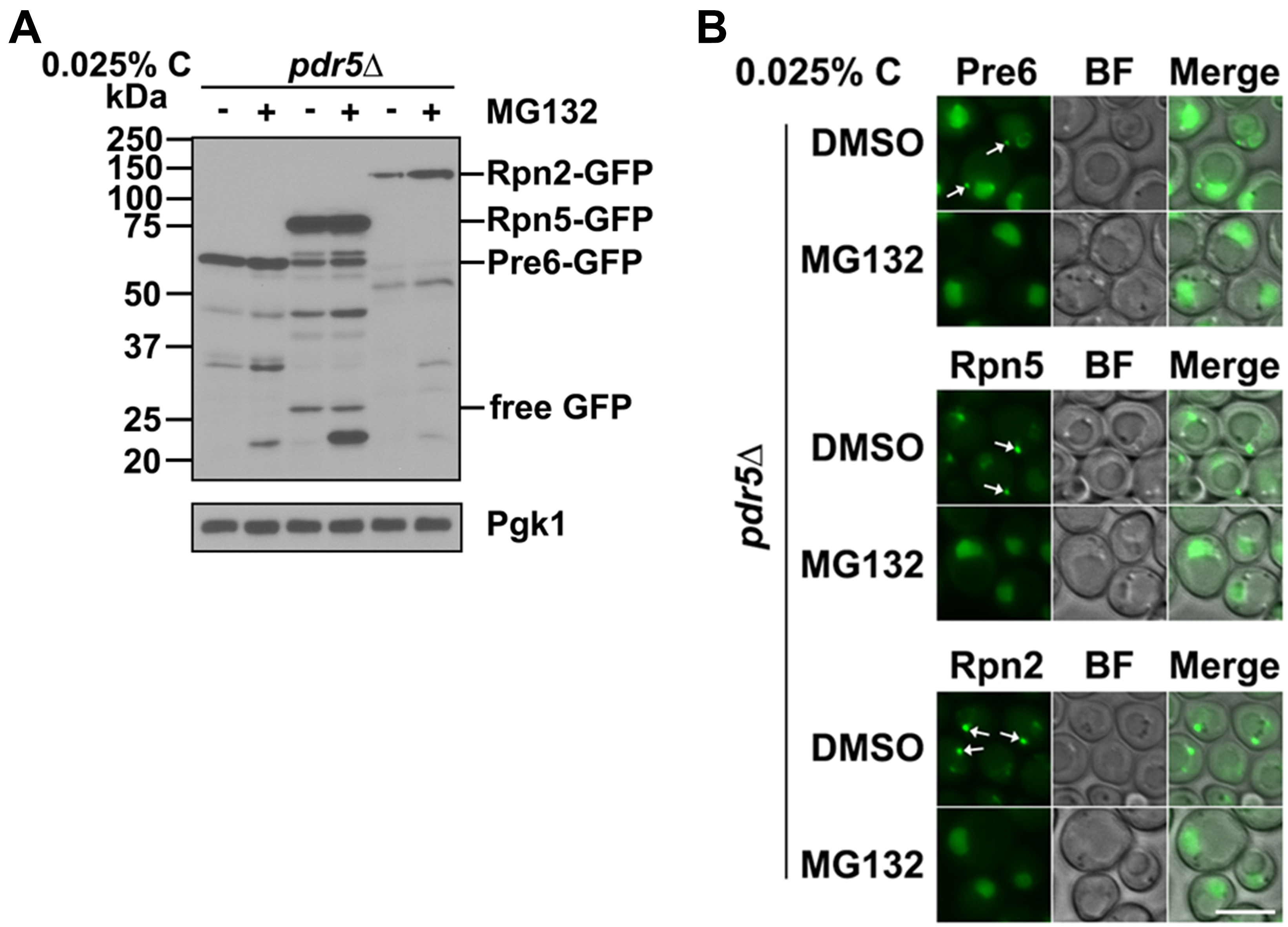

Supplement: S5 Fig — (A) Anti-GFP immunoblot analyses of Pre6-GFP (a CP subunit, α4), Rpn5-GFP, and Rpn2-GFP in pdr5Δ mutant cells. Cells were harvested from cultures in SC medium containing low glucose (0.025% C) containing either DMSO (control) or 50 μM MG132 and grown for ~1 day at 30°C. (B) Epifluorescence images of control and MG132-treated cells from panel (A). White arrows mark PSGs. BF: bright field. Scale bar, 5 μm. (TIF) [file pgen.1008387.s005.tif]

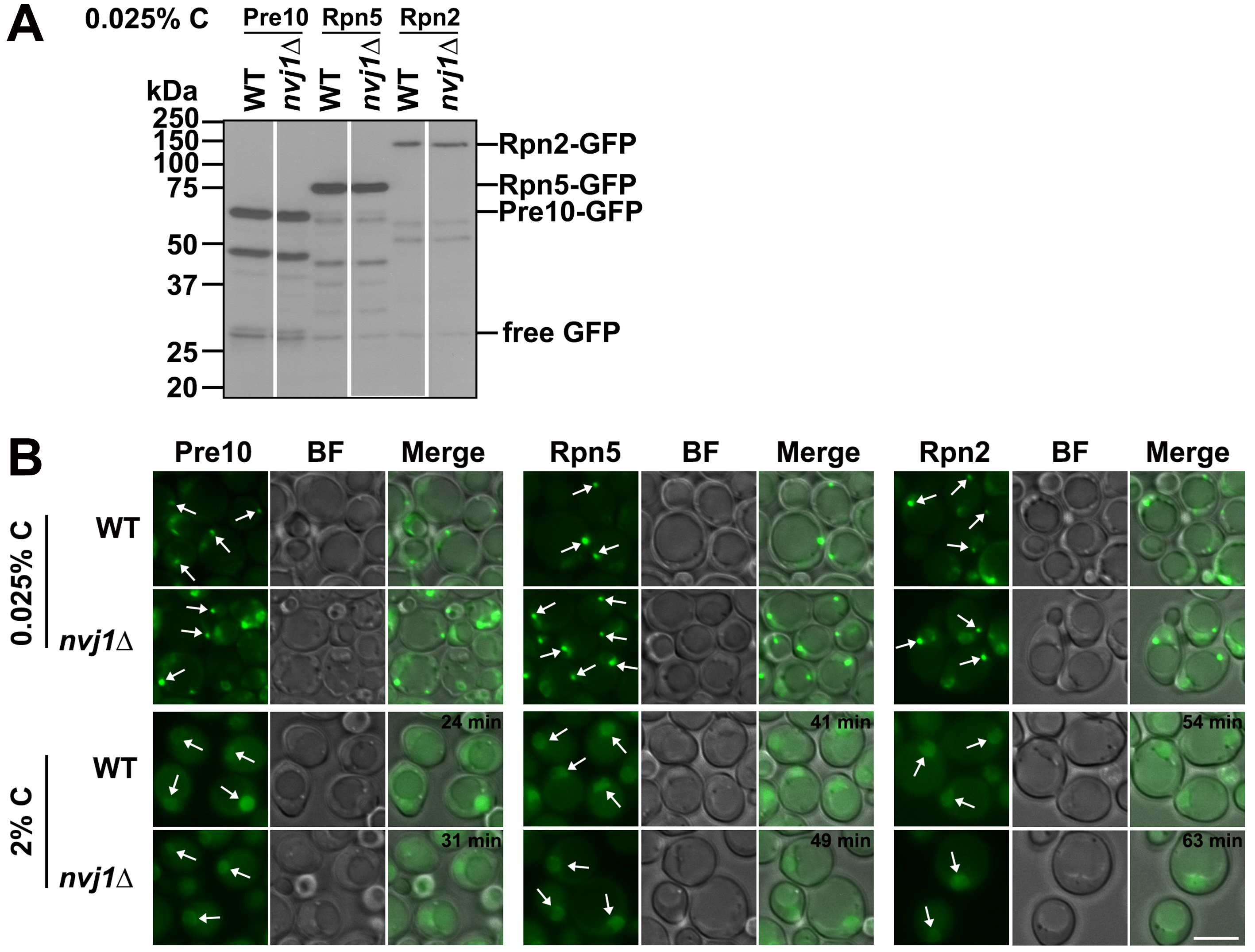

Supplement: S6 Fig — (A) Anti-GFP immunoblot analyses of Pre10-GFP, Rpn5-GFP, and Rpn2-GFP in nvj1Δ mutant cells under low glucose starvation for ~4 days at 30°C. (B) Epifluorescence images of Pre10-GFP, Rpn5-GFP, and Rpn2-GFP in nvj1Δ cells during low glucose starvation and at the indicated times, recovery in 2% glucose. Cells were from figure panel (A). White arrows mark PSGs in the low glucose panels and the nucleus in the glucose refeeding panels. BF: bright field. Scale bar, 5 μm. (TIF) [file pgen.1008387.s006.tif]

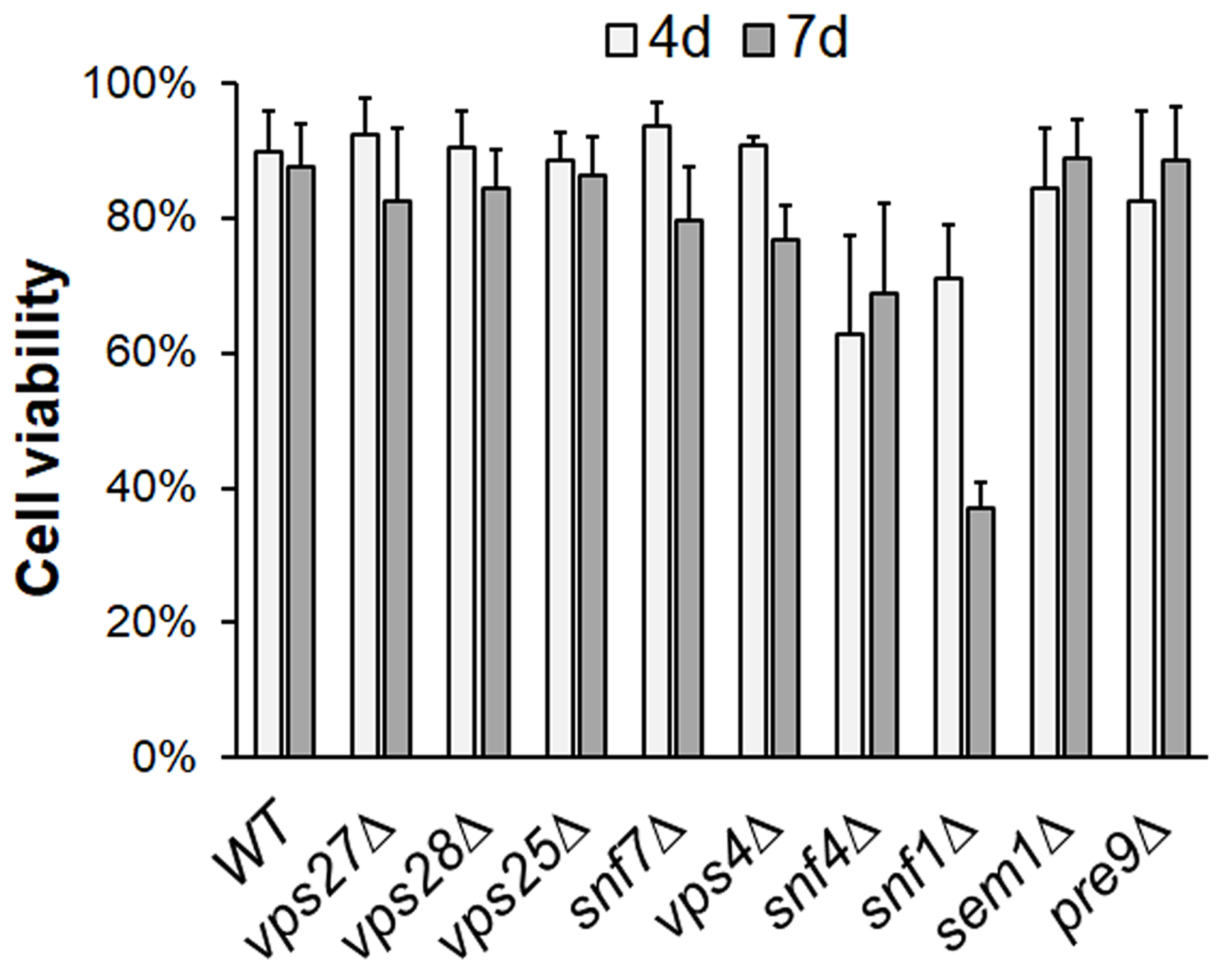

Supplement: S7 Fig — Cell viability assay of WT cells, ESCRT mutants (vps27Δ, vps28Δ, vps25Δ, snf7Δ, vps4Δ), AMPK mutants (snf1Δ and snf4Δ), and proteasome mutants (sem1Δ and pre9Δ) using propidium iodide (PI) staining at day 4 and day 7 in low glucose (30°C). Cells counted (n = 4d, 7d) in WT (775, 668), vps27Δ (904, 644), vps28Δ (706, 652), vps25Δ (761, 715), snf7Δ (835, 659), vps4Δ (636, 654), snf4Δ (843, 685), snf1Δ (842, 608), sem1Δ (713, 731), and pre9Δ (676, 742). (TIF) [file pgen.1008387.s007.tif]
