# Supplementary material for: AMPK regulates ESCRT-dependent microautophagy of proteasomes concomitant with proteasome storage granule assembly during glucose starvation
Source: PLoS Genet. 2019 Nov 18;15(11):e1008387. doi: 10.1371/journal.pgen.1008387 (PMC6886873; doi:10.1371/journal.pgen.1008387)
Supplement: S2 Table — (DOCX) [file pgen.1008387.s012.docx]

**S2 Table. Yeast strains used in this study**

| **Strain** | **Genotype** | **Reference** |
| --- | --- | --- |
| MHY500 | *MATa his3-∆200 leu2-3, 112 ura3-52 lys2-801 trp1-1* | [1] |
| MHY2443 | *MATα his3-∆200 leu2-3, 112 ura3-52 lys2-801 trp1-1 vps4∆::TRP1* | [2] |
| MHY6377 | *MATa his3-∆200 leu2-3, 112 ura3-52 lys2-801 trp1-1 RPN5-yEGFP::HIS3* | This study |
| MHY6614 | *MATa his3-∆200 leu2-3, 112 ura3-52 lys2-801 trp1-1 RPN5-yEGFP::HIS3 pdr5∆::kanMX* | This study |
| MHY7791 | *MATa his3-∆200 leu2-3, 112 ura3-52 lys2-801 trp1-1 RPN2-GFP::kanMX pdr5∆::kanMX* | This study |
| MHY7797 | *MATα his3-∆200 leu2-3, 112 ura3-52 lys2-801 trp1-1 prb1∆::kanMX pep4∆::natMX* | This study |
| MHY8595 | *MATa his3-∆200 leu2-3, 112 ura3-52 lys2-801 trp1-1 NUP49-GFP::his5+ PRE1-mC::natMX* | This study |
| MHY8599 | *MATa his3-∆200 leu2-3, 112 ura3-52 lys2-801 trp1-1 NUP49-GFP::his5+ RPN2-mC::natMX* | This study |
| MHY8602 | *MATa his3-∆200 leu2-3, 112 ura3-52 lys2-801 trp1-1 NUP49-GFP::his5+ RPN5-mC::natMX* | This study |
| MHY8629 | *MATα his3-∆200 leu2-3, 112 ura3-52 lys2-801 trp1-1 NUP49-GFP::his5+ PRE1-mC::natMX did2∆::HIS3* | This study |
| MHY8630 | *MATa his3-∆200 leu2-3, 112 ura3-52 lys2-801 trp1-1 NUP49-GFP::his5+ RPN2-mC::natMX did2∆::HIS3* | This study |
| MHY8632 | *MATa his3-∆200 leu2-3, 112 ura3-52 lys2-801 trp1-1 NUP49-GFP::his5+ RPN5-mC::natMX did2∆::HIS3* | This study |
| MHY8633 | *MATa his3-∆200 leu2-3, 112 ura3-52 lys2-801 trp1-1 NUP49-GFP::his5+ PRE1-mC::natMX vps24∆::HIS3* | This study |
| MHY8634 | *MATa his3-∆200 leu2-3, 112 ura3-52 lys2-801 trp1-1 NUP49-GFP::his5+ RPN2-mC::natMX vps24∆::HIS3* | This study |
| MHY8636 | *MATa his3-∆200 leu2-3, 112 ura3-52 lys2-801 trp1-1 NUP49-GFP::his5+ RPN5-mC::natMX vps24∆::HIS3* | This study |
| MHY8637 | *MATα his3-∆200 leu2-3, 112 ura3-52 lys2-801 trp1-1 NUP49-GFP::his5+ PRE1-mC::natMX vps27∆::LEU2* | This study |
| MHY8639 | *MATα his3-∆200 leu2-3, 112 ura3-52 lys2-801 trp1-1 NUP49-GFP::his5+ RPN2-mC::natMX vps27∆::LEU2* | This study |
| MHY8641 | *MATα his3-∆200 leu2-3, 112 ura3-52 lys2-801 trp1-1 NUP49-GFP::his5+ RPN5-mC::natMX vps27∆::LEU2* | This study |
| MHY8642 | *MATα his3-∆200 leu2-3, 112 ura3-52 lys2-801 trp1-1 NUP49-GFP::his5+ PRE1-mC::natMX vps2∆::LEU2* | This study |
| MHY8643 | *MATa his3-∆200 leu2-3, 112 ura3-52 lys2-801 trp1-1 NUP49-GFP::his5+ RPN2-mC::natMX vps2∆::LEU2* | This study |
| MHY8645 | *MATa his3-∆200 leu2-3, 112 ura3-52 lys2-801 trp1-1 NUP49-GFP::his5+ RPN5-mC::natMX vps2∆::LEU2* | This study |
| MHY8647 | *MATa his3-∆200 leu2-3, 112 ura3-52 lys2-801 trp1-1 NUP49-GFP::his5+ PRE1-mC::natMX vps4∆::TRP1* | This study |
| MHY8649 | *MATa his3-∆200 leu2-3, 112 ura3-52 lys2-801 trp1-1 NUP49-GFP::his5+ RPN2-mC::natMX vps4∆::TRP1* | This study |
| MHY8652 | *MATa his3-∆200 leu2-3, 112 ura3-52 lys2-801 trp1-1 NUP49-GFP::his5+ RPN5-mC::natMX vps4∆::TRP1* | This study |
| MHY9736 | *MATa his3-∆200 leu2-3, 112 ura3-52 lys2-801 trp1-1 PRE10-GFP::HIS3 snf1∆::kanMX* | This study |
| MHY9738 | *MATa his3-∆200 leu2-3, 112 ura3-52 lys2-801 trp1-1 PRE10-GFP::HIS3 snf4∆::kanMX* | This study |
| MHY9744 | *MATa his3-∆200 leu2-3, 112 ura3-52 lys2-801 trp1-1 PRE10-GFP::HIS3 prb1∆::kanMX* | This study |
| MHY9746 | *MATa his3-∆200 leu2-3, 112 ura3-52 lys2-801 trp1-1 PRE10-GFP::HIS3 pep4∆::natMX* | This study |
| MHY9749 | *MATa his3-∆200 leu2-3, 112 ura3-52 lys2-801 trp1-1 PRE10-GFP::HIS3 prb1∆::kanMX pep4∆::natMX* | This study |
| MHY9812 | *MATα his3-∆200 leu2-3, 112 ura3-52 lys2-801 trp1-1 snf4∆::kanMX* | This study |
| MHY9813 | *MATα his3-∆200 leu2-3, 112 ura3-52 lys2-801 trp1-1 snf1∆::kanMX* | This study |
| MHY9848 | *MATa his3-∆200 leu2-3, 112 ura3-52 lys2-801 trp1-1 NUP49-GFP::his5+ PRE1-mC::natMX snf1∆::kanMX* | This study |
| MHY9849 | *MATa his3-∆200 leu2-3, 112 ura3-52 lys2-801 trp1-1 NUP49-GFP::his5+ RPN5-mC::natMX snf1∆::kanMX* | This study |
| MHY9851 | *MATa his3-∆200 leu2-3, 112 ura3-52 lys2-801 trp1-1 NUP49-GFP::his5+ RPN2-mC::natMX snf1∆::kanMX* | This study |
| MHY9856 | *MATa his3-∆200 leu2-3, 112 ura3-52 lys2-801 trp1-1 NUP49-GFP::his5+ PRE1-mC::natMX snf7∆::kanMX* | This study |
| MHY9859 | *MATa his3-∆200 leu2-3, 112 ura3-52 lys2-801 trp1-1 NUP49-GFP::his5+ RPN2-mC::natMX snf7∆::kanMX* | This study |
| MHY9860 | *MATa his3-∆200 leu2-3, 112 ura3-52 lys2-801 trp1-1 NUP49-GFP::his5+ RPN5-mC::natMX snf7∆::kanMX* | This study |
| MHY9866 | *MATa his3-∆200 leu2-3, 112 ura3-52 lys2-801 trp1-1 NUP49-GFP::his5+ PRE1-mC::natMX snf4∆::kanMX* | This study |
| MHY9867 | *MATα his3-∆200 leu2-3, 112 ura3-52 lys2-801 trp1-1 NUP49-GFP::his5+ RPN2-mC::natMX snf4∆::kanMX* | This study |
| MHY9868 | *MATa his3-∆200 leu2-3, 112 ura3-52 lys2-801 trp1-1 NUP49-GFP::his5+ PRE1-mC::natMX vps25∆::kanMX* | This study |
| MHY9870 | *MATa his3-∆200 leu2-3, 112 ura3-52 lys2-801 trp1-1 NUP49-GFP::his5+ RPN2-mC::natMX vps25∆::kanMX* | This study |
| MHY9872 | *MATa his3-∆200 leu2-3, 112 ura3-52 lys2-801 trp1-1 NUP49-GFP::his5+ RPN5-mC::natMX vps25∆::kanMX* | This study |
| MHY9885 | *MATa his3-∆200 leu2-3, 112 ura3-52 lys2-801 trp1-1 NUP49-GFP::his5+ RPN5-mC::natMX snf4∆::kanMX* | This study |
| MHY9943 | *MATα his3-∆200 leu2-3, 112 ura3-52 lys2-801 trp1-1 HSP42-GFP::HIS3 PRE1-mC::natMX* | This study |
| MHY9945 | *MATα his3-∆200 leu2-3, 112 ura3-52 lys2-801 trp1-1 HSP42-GFP::HIS3 RPN2-mC::natMX* | This study |
| MHY9946 | *MATα his3-∆200 leu2-3, 112 ura3-52 lys2-801 trp1-1 HSP42-GFP::HIS3 RPN5-mC::natMX* | This study |
| MHY9948 | *MATα his3-∆200 leu2-3, 112 ura3-52 lys2-801 trp1-1 HSP42-GFP::HIS3 PRE1-mC::natMX snf4∆::kanMX* | This study |
| MHY9950 | *MATα his3-∆200 leu2-3, 112 ura3-52 lys2-801 trp1-1 HSP42-GFP::HIS3 RPN2-mC::natMX snf1∆::kanMX* | This study |
| MHY9953 | *MATα his3-∆200 leu2-3, 112 ura3-52 lys2-801 trp1-1 HSP42-GFP::HIS3 PRE1-mC::natMX snf1∆::kanMX* | This study |
| MHY9956 | *MATα his3-∆200 leu2-3, 112 ura3-52 lys2-801 trp1-1 HSP42-GFP::HIS3 RPN5-mC::natMX snf4∆::kanMX* | This study |
| MHY9958 | *MATα his3-∆200 leu2-3, 112 ura3-52 lys2-801 trp1-1 HSP42-GFP::HIS3 RPN5-mC::natMX snf1∆::kanMX* | This study |
| MHY9967 | *MATa his3-∆200 leu2-3, 112 ura3-52 lys2-801 trp1-1 HSP42-GFP::HIS3 RPN2-mC::natMX snf4∆::kanMX* | This study |
| MHY9989 | *MATa his3-∆200 leu2-3, 112 ura3-52 lys2-801 trp1-1 RPN5-yEGFP::HIS3 vps27∆::LEU2* | This study |
| MHY9992 | *MATa his3-∆200 leu2-3, 112 ura3-52 lys2-801 trp1-1 RPN5-yEGFP::HIS3 vps25∆::kanMX* | This study |
| MHY9993 | *MATa his3-∆200 leu2-3, 112 ura3-52 lys2-801 trp1-1 RPN5-yEGFP::HIS3 vps4∆::TRP1* | This study |
| MHY9998 | *MATa his3-∆200 leu2-3, 112 ura3-52 lys2-801 trp1-1 RPN5-yEGFP::HIS3 snf7∆::kanMX* | This study |
| MHY10011 | *MATa his3-∆200 leu2-3, 112 ura3-52 lys2-801 trp1-1 RPN5-yEGFP::HIS3 snf4∆::kanMX* | This study |
| MHY10014 | *MATa his3-∆200 leu2-3, 112 ura3-52 lys2-801 trp1-1 RPN5-yEGFP::HIS3 snf1∆::kanMX* | This study |
| MHY10015 | *MATa his3-∆200 leu2-3, 112 ura3-52 lys2-801 trp1-1 RPN5-yEGFP::HIS3 vps28∆::kanMX* | This study |
| MHY10018 | *MATa his3-∆200 leu2-3, 112 ura3-52 lys2-801 trp1-1 RPN5-yEGFP::HIS3 vps37∆::kanMX* | This study |
| MHY10021 | *MATa his3-∆200 leu2-3, 112 ura3-52 lys2-801 trp1-1 RPN2-GFP::HIS3* | This study |
| MHY10042 | *MATa his3-∆200 leu2-3, 112 ura3-52 lys2-801 trp1-1 RPN2-GFP::HIS3 atg8∆::hphMX* | This study |
| MHY10044 | *MATa his3-∆200 leu2-3, 112 ura3-52 lys2-801 trp1-1 RPN5-yEGFP::HIS3 atg8∆::hphMX* | This study |
| MHY10047 | *MATa his3-∆200 leu2-3, 112 ura3-52 lys2-801 trp1-1 RPN2-GFP::HIS3 snf4∆::kanMX* | This study |
| MHY10048 | *MATa his3-∆200 leu2-3, 112 ura3-52 lys2-801 trp1-1 RPN2-GFP::HIS3 snf1∆::kanMX* | This study |
| MHY10051 | *MATa his3-∆200 leu2-3, 112 ura3-52 lys2-801 trp1-1 RPN2-GFP::HIS3 snf7∆::kanMX* | This study |
| MHY10052 | *MATa his3-∆200 leu2-3, 112 ura3-52 lys2-801 trp1-1 RPN2-GFP::HIS3 vps37∆::kanMX* | This study |
| MHY10055 | *MATa his3-∆200 leu2-3, 112 ura3-52 lys2-801 trp1-1 RPN2-GFP::HIS3 vps28∆::kanMX* | This study |
| MHY10057 | *MATa his3-∆200 leu2-3, 112 ura3-52 lys2-801 trp1-1 RPN2-GFP::HIS3 vps25∆::kanMX* | This study |
| MHY10058 | *MATa his3-∆200 leu2-3, 112 ura3-52 lys2-801 trp1-1 RPN2-GFP::HIS3 vps27∆::LEU2* | This study |
| MHY10061 | *MATa his3-∆200 leu2-3, 112 ura3-52 lys2-801 trp1-1 RPN2-GFP::HIS3 vps4∆::TRP1* | This study |
| MHY10072 | *MATa his3-∆200 leu2-3, 112 ura3-52 lys2-801 trp1-1 RPN2-GFP::HIS3 prb1∆::kanMX pep4∆::natMX* | This study |
| MHY10073 | *MATa his3-∆200 leu2-3, 112 ura3-52 lys2-801 trp1-1 PRE6-GFP::HIS3 pdr5∆::kanMX* | This study |
| MHY10075 | *MATa his3-∆200 leu2-3, 112 ura3-52 lys2-801 trp1-1 RPN5-yEGFP::HIS3 prb1∆::kanMX pep4∆::natMX* | This study |
| MHY10081 | *MATa his3-∆200 leu2-3, 112 ura3-52 lys2-801 trp1-1 RPN2-GFP::HIS3 pep4∆::natMX* | This study |
| MHY10083 | *MATa his3-∆200 leu2-3, 112 ura3-52 lys2-801 trp1-1 RPN5-GFP::HIS3 pep4∆::natMX* | This study |
| MHY10110 | *MATa his3-∆200 leu2-3, 112 ura3-52 lys2-801 trp1-1 RPN2-GFP::HIS3 atg17∆::hphMX* | This study |
| MHY10114 | *MATa his3-∆200 leu2-3, 112 ura3-52 lys2-801 trp1-1 RPN5-yEGFP::HIS3 atg11∆::hphMX* | This study |
| MHY10115 | *MATa his3-∆200 leu2-3, 112 ura3-52 lys2-801 trp1-1 RPN2-GFP::HIS3 atg11∆::hphMX* | This study |
| MHY10119 | *MATa his3-∆200 leu2-3, 112 ura3-52 lys2-801 trp1-1 RPN5-yEGFP::HIS3 atg17∆::hphMX* | This study |
| MHY10126 | *MATa his3-∆200 leu2-3, 112 ura3-52 lys2-801 trp1-1 RPN2-GFP::HIS3 atg39∆::hphMX* | This study |
| MHY10128 | *MATa his3-∆200 leu2-3, 112 ura3-52 lys2-801 trp1-1 RPN5-yEGFP::HIS3 atg39∆::hphMX* | This study |
| MHY10130 | *MATa his3-∆200 leu2-3, 112 ura3-52 lys2-801 trp1-1 RPN2-GFP::HIS3 prb1∆::kanMX* | This study |
| MHY10131 | *MATa his3-∆200 leu2-3, 112 ura3-52 lys2-801 trp1-1 RPN5-yEGFP::HIS3 prb1∆::kanMX* | This study |
| MHY10147 | *MATa his3-∆200 leu2-3, 112 ura3-52 lys2-801 trp1-1 PRE10-GFP::HIS3* | This study |
| MHY10151 | *MATa his3-∆200 leu2-3, 112 ura3-52 lys2-801 trp1-1 VPH1-GFP::HIS3* | This study |
| MHY10162 | *MATa his3-∆200 leu2-3, 112 ura3-52 lys2-801 trp1-1 PRE10-GFP::HIS3 atg15∆::hphMX* | This study |
| MHY10164 | *MATa his3-∆200 leu2-3, 112 ura3-52 lys2-801 trp1-1 RPN2-GFP::HIS3 atg15∆::hphMX* | This study |
| MHY10166 | *MATa his3-∆200 leu2-3, 112 ura3-52 lys2-801 trp1-1 RPN5-yEGFP::HIS3 atg15∆::hphMX* | This study |
| MHY10169 | *MATa his3-∆200 leu2-3, 112 ura3-52 lys2-801 trp1-1 VPH1-GFP::HIS3 snf1∆::kanMX* | This study |
| MHY10170 | *MATa his3-∆200 leu2-3, 112 ura3-52 lys2-801 trp1-1 VPH1-GFP::HIS3 atg8∆::hphMX* | This study |
| MHY10173 | *MATa his3-∆200 leu2-3, 112 ura3-52 lys2-801 trp1-1 VPH1-GFP::HIS3 vps4∆::TRP1* | This study |
| MHY10175 | *MATa his3-∆200 leu2-3, 112 ura3-52 lys2-801 trp1-1 VPH1-GFP::HIS3 snf4∆::kanMX* | This study |
| MHY10179 | *MATa his3-∆200 leu2-3, 112 ura3-52 lys2-801 trp1-1 PRE10-GFP::HIS3 vps4∆::TRP1* | This study |
| MHY10181 | *MATa his3-∆200 leu2-3, 112 ura3-52 lys2-801 trp1-1 PRE10-GFP::HIS3 vps25∆::kanMX* | This study |
| MHY10182 | *MATα his3-∆200 leu2-3, 112 ura3-52 lys2-801 trp1-1 PRE10-GFP::HIS3 vps27∆::LEU2* | This study |
| MHY10183 | *MATa his3-∆200 leu2-3, 112 ura3-52 lys2-801 trp1-1 PRE10-GFP::HIS3 vps37∆::kanMX* | This study |
| MHY10185 | *MATa his3-∆200 leu2-3, 112 ura3-52 lys2-801 trp1-1 PRE10-GFP::HIS3 snf7∆::kanMX* | This study |
| MHY10187 | *MATa his3-∆200 leu2-3, 112 ura3-52 lys2-801 trp1-1 PRE10-GFP::HIS3 vps28∆::kanMX* | This study |
| MHY10190 | *MATa his3-∆200 leu2-3, 112 ura3-52 lys2-801 trp1-1 PRE10-GFP::HIS3 atg8∆::hphMX* | This study |
| MHY10192 | *MATa his3-∆200 leu2-3, 112 ura3-52 lys2-801 trp1-1 PRE10-GFP::HIS3 atg11∆::hphMX* | This study |
| MHY10193 | *MATa his3-∆200 leu2-3, 112 ura3-52 lys2-801 trp1-1 PRE10-GFP::HIS3 atg17∆::hphMX* | This study |
| MHY10195 | *MATa his3-∆200 leu2-3, 112 ura3-52 lys2-801 trp1-1 PRE10-GFP::HIS3 atg39∆::hphMX* | This study |
| MHY10326 | *MATa his3-∆200 leu2-3, 112 ura3-52 lys2-801 trp1-1 VPH1-GFP::HIS3 atg15∆::hphMX* | This study |
| MHY10328 | *MATa his3-∆200 leu2-3, 112 ura3-52 lys2-801 trp1-1 PRE10-GFP::HIS3 sem1∆::kanMX* | This study |
| MHY10330 | *MATa his3-∆200 leu2-3, 112 ura3-52 lys2-801 trp1-1 RPN2-GFP::HIS3 sem1∆::kanMX* | This study |
| MHY10332 | *MATa his3-∆200 leu2-3, 112 ura3-52 lys2-801 trp1-1 RPN5-yEGFP::HIS3 sem1∆::kanMX* | This study |
| MHY10335 | *MATa his3-∆200 leu2-3, 112 ura3-52 lys2-801 trp1-1 PRE10-GFP::HIS3 pre9∆::hphMX* | This study |
| MHY10336 | *MATa his3-∆200 leu2-3, 112 ura3-52 lys2-801 trp1-1 RPN2-GFP::HIS3 pre9∆::hphMX* | This study |
| MHY10339 | *MATa his3-∆200 leu2-3, 112 ura3-52 lys2-801 trp1-1 RPN5-yEGFP::HIS3 pre9∆::hphMX* | This study |
| MHY10354 | *MATα his3-∆200 leu2-3, 112 ura3-52 lys2-801 trp1-1 PRE1-mC::natMX VPH1-GFP::HIS3 vps4∆::TRP1* | This study |
| MHY10361 | *MATa his3-∆200 leu2-3, 112 ura3-52 lys2-801 trp1-1 RPN5-mC::natMX VPH1-GFP::HIS3 vps4∆::TRP1* | This study |
| MHY10371 | *MATa his3-∆200 leu2-3, 112 ura3-52 lys2-801 trp1-1 RPN2-mC::natMX VPH1-GFP::HIS3 vps4∆::TRP1* | This study |
| MHY10717 | *MATa/α his3-∆200 leu2-3, 112 ura3-52 lys2-801 trp1-1 PRE10-GFP::HIS3 atg8∆::hphMX prb1∆::kanMX pep4∆::natMX* | This study |
| MHY10719 | *MATa/α his3-∆200 leu2-3, 112 ura3-52 lys2-801 trp1-1 RPN5-yEGFP::HIS3 atg8∆::hphMX prb1∆::kanMX pep4∆::natMX* | This study |
| MHY10724 | *MATa/α his3-∆200 leu2-3, 112 ura3-52 lys2-801 trp1-1 RPN2-GFP::HIS3 atg8∆::hphMX prb1∆::kanMX pep4∆::natMX* | This study |
| MHY10720 | *MATα his3-∆200 leu2-3, 112 ura3-52 lys2-801 trp1-1 atg8∆::hphMX prb1∆::kanMX pep4∆::natMX* | This study |
| MHY10711 | *MATα his3-∆200 leu2-3, 112 ura3-52 lys2-801 trp1-1 vps4∆::TRP1 prb1∆::kanMX pep4∆::natMX* | This study |
| MHY10808 | *MATα his3-∆200 leu2-3, 112 ura3-52 lys2-801 trp1-1 atg8∆::hphMX vps4∆::TRP1 prb1∆::kanMX pep4∆::natMX* | This study |
| MHY11059 | *MATa/α his3-∆200; leu2-3, 112; ura3-52; lys2-801; trp1-1; gal2; snf4∆::kanMX; atg8∆::hphMX; pep4∆::natMX; prb1∆::kanMX* | This study |
| MHY11063 | *MATa his3-∆200; leu2-3, 112; ura3-52; lys2-801; trp1-1; gal2; snf1∆::kanMX; vps4∆::TRP1; VPH1-GFP::HIS3; PRE1-mC::natMX* | This study |
| MHY11065 | *MATα his3-∆200; leu2-3, 112; ura3-52; lys2-801; trp1-1; gal2; snf1∆::kanMX; vps4∆::TRP1; VPH1-GFP::HIS3; RPN5-mC::natMX* | This study |
| MHY11068 | *MATα his3-∆200; leu2-3, 112; ura3-52; lys2-801; trp1-1; gal2; snf1∆::kanMX; vps4∆::TRP1; VPH1-GFP::HIS3; RPN2-mC::natMX* | This study |

**Supporting References**

1. Chen P, Johnson P, Sommer T, Jentsch S, Hochstrasser M. Multiple ubiquitin-conjugating enzymes participate in the in vivo degradation of the yeast MATα2 repressor. Cell. 1993;74(2):357-69. doi: 10.1016/0092-8674(93)90426-Q.
2. Amerik A, Sindhi N, Hochstrasser M. A conserved late endosome–targeting signal required for Doa4 deubiquitylating enzyme function. J Cell Biol. 2006;175(5):825-35. doi: 10.1083/jcb.200605134.
