# Supplementary material for: AMPK regulates ESCRT-dependent microautophagy of proteasomes concomitant with proteasome storage granule assembly during glucose starvation
Source: PLoS Genet. 2019 Nov 18;15(11):e1008387. doi: 10.1371/journal.pgen.1008387 (PMC6886873; doi:10.1371/journal.pgen.1008387)
Supplement: Attachment — Submitted filename: Li et al. Response to reviewers.pdf [file pgen.1008387.s013.pdf]

Responses to Review Comments and Questions

We thank all the reviewers for the constructive comments. Below we provide a detailed response to all of the points they have raised.

Comments to the Authors:
Please note here if the review is uploaded as an attachment.

Reviewer #1: PGENETICS-D-19-01412

The work describes an AMPK Snf1- and ESCRT-dependent sorting pathway for micro-autophagy of presumably aberrant proteasomes versus the sequestration of still functional proteasomes into proteasome storage granules (PSGs) during glucose starvation. The work is based on Yeast Cell Biology by using in vivo fluorescence microscopy of yeast cells expressing GFP-labelled proteasomal subunits, electron microscopy and Western blot analysis.
Despite limitations of these techniques the work is interesting and will inspire the scientific community to analyze quality control pathways not only of proteasomes but also of other giant protein complexes which might be recycled or stored in liquid liquid phase condensates depending on AMPK Snf1.

Several points must be addressed before a revision of the paper can be considered.

Major points:

Fig. 1E, immunogold labelling was performed with anti-CP (Enzo #BML-PW9355),
Please show a Western blot of total lysate and purified proteasomes for antibody specificity.

The antibody can specifically recognize multiple alpha subunits of the 20S proteasome. (a) We have tested it in yeast cells by immunofluorescence microscopy (Fig on the right). (b) This commercial yeast anti-20S antibody is produced with immunogen from *Saccharomyces cerevisiae*-derived proteasomes and has been validated for use in detecting CP subunits of yeast proteasomes by immunoblot analyses in previous studies over many years. Please refer to the published immunoblot results, such as Figs 1h and 3b in Rousseau et al. Nature (2016); Fig 4B in Chien et al., J Biol Chem. (2013), and Fig 1d in Howell et al., Sci Rep. (2019).

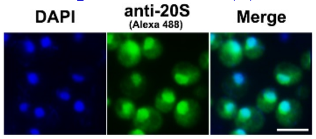

EM images do not show gold particles accumulating in a spherical ‘organelle’ with ~ 500 nm diameter. Achievements over previous EM images showing clear clusters of gold particles with ~ 100-200 nm diameter must be discussed (Laporte et al. 2008, JCB). Did GFP antibodies result in similar immuno gold proteasome localization ?

The reviewer is correct in pointing out the size difference. The gold particles accumulate in clear cytoplasmic clusters in our electron micrographs as well. The shape was not spherical, which was true in both our micrographs and previous EM images in Fig 1F in Laporte et al. J Cell Biol. (2008). As for the size, we think this may be due to several factors. (a) Different culture medium and glucose starvation time (4 d in low glucose medium in our case versus 4 d in YPD medium); this is likely the major contributing factor. We noticed that PSGs became larger, as indicated by the increased fluorescence intensity of PSGs, from day 1 to day 4 under glucose starvation conditions. This is consistent with the PSG formation kinetics that had been described in Fig S3 in Laporte et al. (2008). Therefore, PSGs still accumulate proteasomes as the culture ages. (b) Different protocols for immunogold EM; fixation and processing differences could affect ‘organelle’ size.

In our hands, the GFP antibodies did not work for immunogold EM labelling of GFP-tagged proteasome subunits. We tested two different anti-GFP antibodies: polyclonal rabbit anti-GFP (Life

Technologies, catalog # A11122) and polyclonal chicken anti-GFP (Life Technologies, catalog # A10262). Both GFP antibodies did not successfully label PSGs in WT cells, instead showing a lot of non-specific uptake in the outer membrane of the yeast cells.

Fig. 6E. One to two gold particles on an EM micrograph are not statistically relevant for making sweeping claims. Please provide quantification for justification.

We agree with the reviewer's suggestion. We now have quantified our EM images in three aspects: (1) the percentage of cell sections with the indicated structures (autophagic bodies (ABs) and/or microautophagy vesicles) visible in the vacuole; (2) the percentage of the ABs or vesicles with gold particle staining; and (3) the percentage of intravacuolar gold particles that associated with the indicated structures in the cells. We added three new figure panels as Fig 6F, 6G, and 6H in the new Fig 6, and also included new strain that lacked AMPK in addition to the autophagy factor Atg8 (*atg8*Δ *snf4*Δ *pep4*Δ *prb1*Δ) in the new Fig 6; we were excited to see that these were almost devoid of either ABs or microautophagy vesicles, as we had seen with *atg8*Δ *vps4*Δ *pep4*Δ *prb1*Δ, arguing that AMPK signaling functions specifically in the microautophagy pathway.

To reflect these changes, we modified the text as follows.

Lines 328-332: "Strikingly, by anti-CP immunogold electron microscopy, large numbers of membrane vesicle-like structures accumulated in the vacuole in ~82% of *atg8*Δ *pep4*Δ *prb1*Δ cell sections (Fig 6D and 6F); ~29% of vesicle-containing cell sections were marked with gold beads (Fig 6G). Roughly 81% of the intravacuolar gold beads marking proteasomes were on the edges of the membrane vesicle-like structures in *atg8*Δ *pep4*Δ *prb1*Δ cells (Fig 6D and 6H)."

Lines 334-338: By contrast, in glucose-limited *vps4*Δ *pep4*Δ *prb1*Δ cells, which lack the full ESCRT pathway and therefore microautophagy, CP-labeled autophagic bodies (ABs) were observed in the vacuole lumen instead; ~77% of the cell sections included ABs (Fig 6E and 6F), and in these sections, ~63% were labeled with anti-CP gold beads, of which ~89% marked ABs (Fig 6D, 6G, and 6H).

Lines 340-345: Moreover, the microautophagic vesicles and ABs were induced simultaneously as both structures were observed in ~83% of *pep4*Δ *prb1*Δ cell sections, while neither was visible in ~96% of *atg8*Δ *vps4*Δ *pep4*Δ *prb1*Δ and ~84% of *atg8*Δ *snf4*Δ *pep4*Δ *prb1*Δ cell sections (Fig 6D and 6F). These findings support the hypothesis that AMPK specifically regulates ESCRT-dependent microautophagy of proteasomes in low glucose conditions.

p.11, 232 Where do proteasomes localize in vps4 snf1 double null mutants ?

We now have looked at proteasome subunits Pre1-mC, Rpn5-mC, and Rpn2-mC in *snf1*Δ *vps4*Δ cells expressing Vph1-GFP under low glucose conditions and added the results to Fig 3B and 3C in the new Fig 3. The proteasome localization pattern was very similar to that in *snf1*Δ cells (Fig 1C). Interestingly, we observed reduced colocalization of proteasomes and Vph1-GFP in *snf1*Δ *vps4*Δ as compared to *vps4*Δ cells. This points to a specific role for AMPK in promoting ESCRT-dependent trafficking of proteasomes to the vacuole; Vph1-GFP already resides in the vacuole membrane and may therefore not require AMPK.

p. 11, 228 What is the evidence that Vph1-GFP is suited as a marker for the class E-like compartment ? Would Cps1 be a better choice to identify this dot at the vacuole as a class-E like compartment (jcs.biologists.org/content/125/21/5208) ? Fig. 3B requires a statistical analysis for the coincidence of Vph1 with proteasomes in the same spot at the vacuole. Moreover, V-type ATPase (Vph1 subunit) disassembles with glucose deprivation (Martinez and Munoz, 2008). Is Vph1 then invaginated into the vacuole at the class E-like compartment for lysosomal degradation ? The rational for choosing Vph1 as class E marker is unclear.

We agree that Cps1 is a validated marker for class E compartments of ESCRT mutants. Nevertheless, Vph1-GFP is also a suitable marker for these compartments. Previous studies have shown that Vph1 accumulates within class E compartments in ESCRT mutants, as demonstrated by immunofluorescence microscopy in Piper et al. J Cell Biol. (1997) and also by GFP tagging in

MacDonald et al. Dev. Cell (2015). Vph1 is transported from the Golgi to the vacuole via the same used by Cps1 (Piper et al. J Cell Biol. 1997). Moreover, a recent study has demonstrated ESCRT-dependent microautophagy using Vph1-GFP as a substrate (Oku et al. J Cell Biol. 2017), but the mechanistic roles of ESCRTs in Vph1 degradation are still unknown.

We now have quantified Fig 3B and added the quantification result as Fig 3C in the new Fig 3.

p. 16, 342-345. To create cells bearing large numbers of misassembled or abnormal proteasomes…how is this possible, since after 4 days growth to stationary phase the new synthesis of proteasomal precursor complexes is stalled. Will all proteasomes in sem1 null and pre9 null be dysfunctional, thus not sequestered into PSGs ? Did you also analyze ump1 null mutants with severe defects in proteasome assembly ? They show giant PSG formation. Why are aberrant proteasomes in sem1, pre9 and ump1 null not located in IPODs, if only functional proteasomes are worth to be sequestered into PSGs ?

Proteasomes assembled prior to glucose deprivation would be expected to make up the majority of the particles seen. Although all the proteasomes in these two mutants will lack the deleted subunit, the degree of dysfunction might vary as both cause assembly defects in addition to altering the properties of the mature particles. Proteasomes separate into RP and CP components under carbon starvation (Marshall et al. eLife. 2018). Normal RPs could still be forming PSGs in *pre9*Δ cells when CP is abnormal, while no PSGs were formed in *sem1*Δ cells as shown in our study. This is consistent with previous studies with other defective lid subunits. Regarding the *ump1* mutant, we assumed the reviewer is talking about Fig 3 in Saunier et al. PLoS One (2013) in which they showed PSGs of Rpn5-GFP in *ump1*Δ cells. We examined PSG formation and dissipation in *ump1*Δ cells. The results showed that irreversible proteasome granules of Pre10-GFP were formed in *ump1*Δ cells (Fig on the right), while PSG formation from Rpn5-GFP or Rpn2-GFP was significantly inhibited, with a reduction of ~53% and 68% as compared those in WT cells, respectively. One thing we noticed was a very severe synthetic growth defect of Pre10-GFP and *ump1*Δ; it took about 5-6 days for this strain to grow just a small amount. By contrast, only a modest growth defect was observed with Rpn5-GFP or Rpn2-GFP in *ump1*Δ, which was comparable to *ump1*Δ on its own.

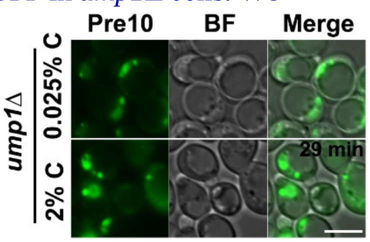

p. 50, 1067 What is an irreversible PSG ? Per definition PSGs are reversible and motile. Are irreversible PSGs converted into IPOD ?

We agree that PSGs are reversible and that our nomenclature was confusing. We have replaced "irreversible PSGs" with "irreversible [proteasome] aggregates" throughout the text. In AMPK mutants, CP-containing irreversible proteasome aggregates mostly converted into IPODs (or became terminal PSGs) as the cultures aged.

p. 20, 452 'adjacent to the vacuolar membrane during PSG assembly' is misleading…Laporte et al. 2008 (Fig. 2B) clearly showed that PSGs arise at the NE, when proteasomes exit the nucleus during the transition from proliferation to quiescence.

We thank the reviewer for pointing this out. To clarify, initially, proteasome dots arise near the NE, release from NE and then move to the perivacuolar region for further assembly or maturation. In that paper, they also observed that PSGs became larger with the length of culture. The perivacuolar PSGs may undergo proteasome quality control or maturation, at the interface of PSGs and the vacuolar membrane. We consider this process as a part of PSG assembly. Our observations of PSG formation were also consistent with previous results described in Peters et al. J Cell Sci. (2016).

Figs. 1 to 3 Fluorescence microscopy studies need to be quantified, n≥200 cells, also for supplementary Figures.

We agree and have now quantified these microscopy data. We now have added the quantification results as Fig 1E in the new Fig 1, Fig 2C in the new Fig 2, and Fig 3C in the new Fig 3, as well as Fig S2C in the new Fig S2 in the supporting information section.

Fig. 2B. The vacuolar membrane can hardly be seen by non-experts. The 4X magnifications are dark grey pixels on a light grey background with no clue how the vacuolar membrane was assigned by the doted white line. The vacuolar marker FM4-64 or MDY-64 should be used to visualize the vacuolar membrane in red or yellow, while proteasome dots are green.

We tested FM4-64 as a marker in WT and ESCRT mutants (Fig on the right). The giant bright perivacuolar dots disturb or override the purpose of using FM4-64 for vacuolar membrane staining in ESCRT mutants. Therefore, we thought it would be a better way to annotate the approximate vacuolar membrane boundary with dotted white line as shown in Fig 2B. We agree with that the vacuolar membrane in the original Pre10-GFP panel was not clear, so we replaced the Pre10-GFP panel with clearer ones under bright field.

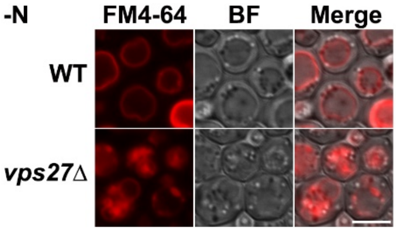

Figs. 2A, 3A and 5A. Are the Western blots representative for three biological replicates?

We usually keep two genetic isolates with different mating types for each strain genotype in stock, and these Western blots were done with both isolates and had been repeated at least 3 or 4 times with these isolates. These data are highly reproducible.

The proteasome dots at the vacuole seem to be close to the nucleus. Did you also check for piecemeal microphagy by using Nvj1 as marker ?

We thank the reviewer for this insightful suggestion. We now have checked proteasome fragmentation and PSG formation and dissipation in *nvj1*Δ cells, which are incapable of executing piecemeal microautophagy. We added the results to S6 Fig in the supporting information section. This mutation did not cause any defects as compared to WT cells.

Fig. S5. According to Marshall & Vierstra the addition of MG132 for 4 h induces proteasome dots in yeast (eLife 2018). Kaganovich et al. (2008 Nature) observed JUNQ upon MG132 exposure. JUNQ and PSGs are both reversible and may describe the same membraneless organelle. How do the authors explain the discrepancy between these observations ? Was MG132 after 1 day treatment still effective ?

The two aforementioned papers described two distinct cytoplasmic aggregates: IPOD and JUNQ. The proteasome dots were terminally sequestered to IPODs, instead of forming PSGs, in Marshall et al. eLife (2018). As shown in the figure legend of Fig 1G in Marshall et al. eLife (2018), the *erg6*Δ cells were treated with MG132 in +N+C medium for 24 h to inactivate proteasomes. The resulting proteasome aggregates were converted into IPODs, because the formation of proteasome aggregates was dependent on the Hsp42 chaperone. This was a repeat of their previous results (Marshall et al Cell Rep. 2016); whether the proteasome dots formed under this condition were reversible or not had not been shown. In Kaganovich et al. Nature. (2008), the *pdr5*Δ cells were treated with MG132 for just 1 h at 37°C or 30°C and thus induced perinuclear ER re-localization of proteasomes, i.e., what they named JUNQ. We think the different MG132 incubation times are the major factor that caused the different accumulation of these two distinct aggregates. Therefore, we think there is no discrepancy between these observations.

MG132 was still effective after 1 day treatment. Previous studies have demonstrated that MG132 could still effectively inhibit proteasome activity in yeast cells after 1 day treatment, as shown in Fig 6C in Marshall et al. eLife (2018). In our experiments, MG132 could still effectively inhibited cell growth and PSG formation under low glucose starvation conditions (S5B Fig).

Minor points:

p. 3, 55; p.4, 80 What is the evidence for phase separation of PSGs ?

We do not have direct evidence for the idea of phase separation of PSGs, but this has been suggested several times in the literature given their rapid reversibility and membraneless nature. However, we have removed all references to phase separation in the revised manuscript.

It should be mentioned that Snf1 was also identified in a previous high-content screen for PSG formation, Gu et al. 2017 Fig. 1B.

We thank the reviewer for pointing out this missing citation. We have now cited the paper.

p.10, 222 S2A Fig. turn around

We followed the figure citation format of the supporting information as instructed by the journal (which we agree is unusual!).

p. 12, 262-265 unclear longwinded sentence

We now have revised this sentence to "This suggests that a fraction of the CP-containing foci in AMPK mutants, which still colocalized with IPODs after glucose recovery, had either become 'terminal PSGs' or matured into IPODs. The expected difference between these two structures would be the greater compositional complexity of IPODs."

p. 14, 297-299 Is the vacuole acidification dependent of the cytoplasmic pH and the amount of glucose in the cytoplasm ? If the vacuolar pH optimum is not reached, the vacuolar proteases may not be fully active leading the proteasome fragmentation. Please comment.

We thank the reviewer for this insightful comment and agree that the activity of vacuolar proteases could potentially be related to proteasome fragmentation patterns. The V-ATPase complex is affected by cytosolic pH and glucose concentration; specifically, glucose starvation reduces cytosolic pH and cause the disassembly of V-ATPase (Dechant et al. EMBO J. 2010). However, since vacuolar acidification is essential for breaking down autophagic bodies (Nakamura et al. J Biochem. 1997), we checked glucose-starved WT and *pep4*Δ *prb1*Δ cells by immunogold EM to check for any residual degradation products in the vacuole. The result indicated that the two major vacuolar proteases could efficiently degrade the substrates in the vacuole as the vacuole was clear in WT cells (Fig on the right), whereas autophagic bodies and membrane vesicles accumulated in *pep4*Δ *prb1*Δ cells (Fig 6D). This suggests that the activity of vacuolar proteases was not significantly affected under glucose starvation conditions.

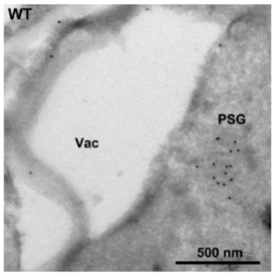

p. 19, 413 as true for blm10 null mutants (Weberruss et al. 2013)

We now have mentioned *blm10*Δ as in lines 420-422: "This supports the inference that proteasomes separate into CP and RP complexes during glucose starvation, and further suggests that PSGs assemble from RP complexes alone in cells expressing a defective CP, analogous to previous observations in *blm10*Δ cells [15]."

p. 21 last paragraph about LDs is distracting and can be deleted

We understand the reviewer's suggestion, but we hope to keep this paragraph for discussing our proposed mechanistic roles of AMPK in regulation of ESCRT-dependent microautophagy of proteasomes under glucose starvation conditions. In response, however, we have significantly reduced the length of this paragraph.

p. 33, 793-794 repetition of sentence before

We deleted the repeated sentences: “PSG assembly is largely disrupted in the mutants” and “Defective nuclear reimport of proteasomes was also observed in the ESCRT mutants” in the Fig S1 legend.

p. 38, exact number of cells can be replaced by n≥200

We thank the reviewer for this suggestion, but we wish to keep the exact number of cells counted in the quantification assay to accurately reflect the sample sizes being analyzed in the experiments.

In the discussion I miss a clear statement about the authors’ new findings with regard to Ben-Aroya’s paper (ref. 17) who already proposed a sorting mechanism of PSGs versus IPODs at the endosome. Own achievements will not be diminished, if previous work from other groups is honestly acknowledged. There are only a couple of papers on PSG which we should know by heart and cite properly.

We completely agree with this comment. We have cited this paper frequently in our manuscript and note their having connected PSGs with IPODs in the penultimate paragraph of our Discussion.

Reviewer #2: In this manuscript, Li et al. study the dynamics of proteasome assemblies and proteasome quality control in the face of various nutrient stresses, and the contribution of yeast AMPK and ESCRT complexes in this regulation.

Summary and general assessment of the manuscript

The authors use a high-content imaging screening approach to look at proteasome storage granules (PSG) dissipation upon glucose refeeding. The fact that both AMPK, a well-known energy-sensing system, and ESCRT proteins, known to be involved in the protein targeting to the vacuole through various processes such as MVB sorting, microautophagy, etc, are identified is very interesting and indeed merits further consideration.
Then, the authors move on to the study of proteasome macroautophagy induced under nitrogen limitation. The authors describe that ESCRT proteins are required for this process, and as such, this is reminiscent of ESCRT-dependent microautophagy described in the same conditions (Ref 22). Proteasome subunits localize to perivacuolar dots in ESCRT mutants but whether they are of endosomal origin is not addressed; similarly, the mechanism by which ESCRT may help proteasome degradation remains unexplored at this stage.

We thank the reviewer for these interesting questions. At this stage, we do not have definitive data to show whether the perivacuolar dots of proteasomes in ESCRT mutants originated from endosomes. However, in our time lapse data with *atg8*Δ *pep4*Δ *prb1*Δ cells suggest that proteasomes are being taken up directly in invaginating tubules and vesicles at the vacuole surface. Our immune-EM data also support this inference. Such structures have previously been described for microautophagy at the vacuole (Ref. 22).

As for the roles of ESCRTs in vacuole-mediated proteasome degradation, we proposed that ESCRTs may help to seal proteasome-containing autophagosomes during macroautophagy, as has been shown previously, and a similar role could be expected during vacuolar membrane vesiculation during microautophagy, which we have added to the Discussion. Further investigation is needed to understand how ESCRTs promote proteasome degradation under these conditions.

Building upon previous work by the Ohsumi lab (Ref 41), the authors draw similarities between proteasome degradation and autophagy induction during C starvation. Similar to what has been described, they observe that switching cells to low glucose (0.025% in their case) triggers

proteasome degradation, whereas this is barely the case for the no-glucose condition (in which there are no major changes in the abundance of FL proteins, except maybe for Rpn2). Again, proteasome degradation requires most of the ESCRT components tested as observed under N starvation, but this time it also involves AMPK, in line with the fact that autophagy induction in these conditions is AMPK dependent (Ref 41).

Our data indicate that microautophagy, and not just macroautophagy, is important for proteasomal degradation under carbon limitation conditions. The difference in microautophagy –as read out by fragmentation of proteasome subunits– between no glucose and 0.025% glucose is surprising but highly reproducible and in line with what had been reported in Ref. 41 (Ref. 42 now in the revised manuscript), although the latter paper only investigated macroautophagy. Our data link AMPK primarily to the microautophagy mechanism.

It should also be noted that this degradation is not complete - only partial degradation occurs, and the amount of free GFP is overall comparable in the WT and the mutants tested. However, there are differences in the proteolytic fragments likely resulting from incomplete digestion, which the authors use a readout. This partial digestion, or as the authors put it, this "fragmentation", appears to occur before internalization in the vacuole since proteasome subunits localize at the periphery of the vacuolar membrane in the aberrant "class E" compartments observed in ESCRT mutants. As shown later in the manuscript, this fragmentation is independent of many autophagy genes, but still required vacuolar proteases, which is a bit puzzling.

We observed reduced partial proteasome fragmentation in ESCRT mutants (Fig 3). In line with our microscopic observations in ESCRT mutants, proteasomes were trapped at the perivacuolar region, most likely at class E-like compartments, and could not be efficiently delivered to the vacuole for degradation, and thus present reduced fragmentation. Blocking all macroautophagy with *atg8*Δ blocks the generation of free GFP (Fig 5A), providing a readout of this pathway, whereas blocking microautophagy with ESCRT or AMPK mutants, prevents the partial digestion of proteasome subunits. These immunoblot results thus distinguish two autophagic pathways for proteasome targeting into the vacuole during carbon limitation, which is consistent with our immuno-EM results. Specifically, membrane tubule- and vesicle-associated proteasomes were observed in the vacuole in *atg8*Δ *pep4*Δ *prb1*Δ cells and with ABs in *vps4*Δ *pep4*Δ *prb1*Δ cells (Fig 6D).

Another perivacuolar compartment that is important for PSG biology is the protein deposit compartment, IPOD as defined by the marker Hsp42. Co-localization analyses show a strikingly increased association of Pre1 with the IPOD in AMPK mutant, suggesting that AMPK normally prevents association of PSG to the IPOD upon switching cells to low glucose conditions. This is an important observation because Snf1 is inhibited by glucose, so I was initially puzzled as to how Snf1 activity could be solicited when glucose is added back. As the authors suggest, AMPK may be required to salvage proteasomes away from IPOD structures or "terminal" PSGs.

A strong point is the author's efforts to understand the physiological function of these assemblies & associated regulation. The authors hypothesize that aberrant proteasomes may be generated by C stress - a hypothesis that is not further discussed - and that these could be targeted by microautophagy. Genetic or chemical perturbation of proteasome integrity indeed correlate with an alteration of PSG formation and increased proteasome degradation.

We detected proteasomes disassembled into CP and RP under glucose starvation conditions by native gel analyses (data not shown); this is consistent with a previous study using long-term incubation in stationary phase (Bajorek et al. Curr Biol. 2003). Meanwhile, a basal colocalization rate between proteasome subunits and IPODs was observed in WT cells (Fig 4). Previous studies have suggested that IPODs serve as cellular compartments for sequestering the misassembled proteasome subunits (Peters LZ, et al. PLoS Genet. 2015). Taken together, our data suggest that aberrant proteasomes are most likely being produced under glucose starvation conditions, agreeing well with our results in Fig 7 and S5 Fig.

Overall, I found the topic very interesting with a nice set of supporting observations which are technically very well performed. A limitation is that the study is quite broad, making interesting connections between PSG dynamics, quality control and proteasome degradation, AMPK signaling, ESCRT biology, N- starvation and C-starvation or even C removal, autophagy, etc. and consequently many questions remain unanswered. In the end, key questions such how AMPK controls PSG dissipation, how ESCRT "collect" proteasomes for their degradation and why all ESCRT subunits are not always required, how some proteasome subunits escape this regulation, to what do proteasome "fragmentation" and "full degradation" correspond, remain unanswered. Most of these questions are mentioned in the discussion with possible hypotheses, and I understand that each of these questions could be a study in itself, but I was left under the impression that the manuscript is a collection of many observations and is sometimes too superficial.

I am also not sure that the order of appearance of the findings in the manuscript is optimal. The reader is carried from Glc refeeding to N starvation, then to C starvation, then back to Glc refeeding, then back to autophagy, etc. and I think that shuffling some parts could generate a more readable manuscript. Of course, this is just my opinion, but maybe the authors can give it a thought.

We thank the reviewer for this suggestion. We have struggled a little with the sequence, but in the end feel it is best in the current order. The key component connecting each section is the PSG. We raised the question first in screening cellular components involved in PSG dissipation during glucose refeeding, and found AMPK and ESCRTs are involved in PSGs formation and dissipation. To explain these observations, we set out to see how proteasome homeostasis is regulated by autophagy and PSGs in AMPK and ESCRT mutants, in part inspired by the paper by Marshall et al. eLife (2018). We then confirmed that AMPK regulates proteasome degradation and IPOD association of PSGs, and this led to the discovery of ESCRT-dependent microautophagy of proteasomes during glucose limitation.

Specific comments.
-Fig 1, B and D: the use of profiles from one cell is questionable and doesn't add much. These profiles can vary a lot depending on how they are drawn across the nucleus and in the end, I think the picture is more informative. Same for S1, B.

We thank the reviewer for this suggestion and agree with the variations of line scan profiles. We selected representative cells for the line scan profiles, and hope to keep the line plots together with the microscopic images to give readers a general idea of the defective nuclear reimport phenotype observed in ESCRT and AMPK mutant cells.

-p7 l.137: From the results section, it is not clear how the authors proceed to "refeed" cells with glucose and see PSG dissipation - this could be better explained.

We thank the reviewer for pointing out this unclear description. We have now added more detail in the materials and methods in lines 532-539: "To uncover mutants that interfered with PSG dissipation and nuclear reimport of proteasomes upon addition of glucose, synthetic defined medium containing glucose was added in the second wash. Plates were then transferred into an automated inverted fluorescent microscopic scan^R system (Olympus) using a swap robotic arm (Hamilton Robotics). The scan^R system is designed to allow autofocus and imaging of plates in 96-well format. Images of cells were recorded ~10 min following glucose replenishment at 24°C using a 60× air lens (NA0.9) and with a cooled charge-coupled device camera (ORCA-ER; Hamamatsu Photonics)."

We also added a new subsection "Construction of an Rpn5-GFP integrated yeast deletion library" in the materials and methods to describe the Rpn5-GFP integrated yeast deletion library, which was published in Peters et al. J Cell Biol. (2013).

-The description of the results of the high-content imaging screen is vague – the precise number of hits is not disclosed, neither is the list. The authors mention "enrichment" but no values are given.

We thank the reviewer for pointing out the missing information and unclear description. We now have added a list of hits as S1 Table in the supporting information section and described the exact number of hits in the text: "The screen identified 198 potential hits (S1 Table)." We noticed a couple of hits were duplicates and thus removed. By "enrichment", we meant that multiple hits from a same pathway were found, we revised this to read "multiple hits from two conserved cellular machineries, AMPK and the ESCRT machinery."

-It came as a surprise that ESCRT proteins display defects in PSG formation, a finding that was not reported in the previous paper (Ref 16) which identified genes involved in PSG formation, also using Rpn5 as a proxy. Is there something I didn't get, or could the authors elaborate?

We used different protocols for yeast genetic screening. In Ref 16 of our original manuscript (Peters et al. 2015), a collection of temperature-sensitive mutants were screened for chromosomal instability, not for PSG formation. But in another paper from the same group (Peters et al. J Cell Biol. 2013), listed as Ref 14 in our original submission, they grew cells in minimal medium to log phase and then looked for abnormal localization of Rpn5-GFP. By contrast, we set up the screening: cells were grown ~3 days in synthetic medium lacking glucose, and then looked at Rpn5-GFP localization at ~10 min after glucose refeeding of the starved-cells. We note that Vps24 (a component of ESCT-III) was identified as a hit in another independent study when yeast cells were grown in YPD medium for 5 days, (Gu et al. Mol Biol Cell. 2017); we have cited this in the revised manuscript.

- p8, l.175: the references cited concern mammalian cells, very few studies link AMPK with autophagy in yeast.

We thank the reviewer for suggesting this accurate description and now have written "Prior work showed that AMPK plays a direct role in promoting macroautophagy in mammalian cells in response to energy deprivation."

We agree that the role of AMPK in autophagy in yeast is not well understood. A previous study showed Snf1 is required for macroautophagy induction under different conditions, based on the substrate GFP-Atg8 (Adachi et al. J Biol Chem. 2017). We now have shown Snf1 is also involved in microautophagy. Further investigations are needed to reveal the mechanistic roles of AMPK in different autophagy pathways.

- Fig. 3B: Do proteasome subunits accumulate in the class E compartment in vps37Δ mutant? If yes, then how could Vps37 be dispensable?

We observed less accumulation of perivacuolar proteasomes at the class E-like compartment and milder defects on PSG formation and dissipation in *vps37*Δ cells as compared to other ESCRT mutants. We quantified the abnormal perivacuolar proteasomes in *vps37*Δ cells and added the result to S2C Fig in the new S2 Fig in the supporting information section. The results suggest that ESCRT-I components do not contribute equally to proteasome degradation and trafficking, perhaps due to different mechanistic roles of ESCRT-I components (Vps23, Vps28, Vps37, Mvb12).

- Fig 6D - why is it that the proteasome subunits are now in the vacuolar lumen in the vps4Δ background, whereas they were perivacuolar by fluorescence microscopy (3B). How representative are these images?

Macroautophagy-dependent proteasome degradation was not inhibited in *vps4*Δ *pep4*Δ *prb1*Δ mutant cells in low glucose conditions, but the two major vacuolar proteases that degrading proteasomes in the vacuole were deleted, so we could observe the macroautophagy dependent proteasome trafficking in the vacuole in this mutant (Fig 6D). Similarly, ESCRT-dependent microautophagy of proteasomes was inhibited in *vps4*Δ mutant cells in low glucose conditions, and vacuolar proteases can degrade ABs and

proteasomes in the vacuole, so microautophagy-dependent proteasomes accumulated in the perivacuolar region in this mutant (Fig 3B).

The fluorescence microscopy and EM images were representative of the general phenotypes we observed in the mutant cells. We have now quantified the fluorescence microscopy and EM images. We added the quantification results as Fig 3C in the new Fig 3 and three figure panels as Fig 6F, 6G, and 6H in the new Fig 6. We also included an AMPK control mutant *atg8*Δ *snf4*Δ *pep4*Δ *prb1*Δ in the new Fig 6. The new text has been modified to take note of these numbers.

- I am wondering how the authors can be sure that the proteolytic fragments observed are degraded in vivo and not during protein preparation. The authors use alkaline lysis, which may not be as stringent regarding endogenous protease activity as is cell lysis in TCA. Have the authors considered this?

We thank the reviewer for pointing out this concern. To confirm the in vivo partial digestion of proteasome subunits, we tested four different protein extraction methods with the same amount of cells (1 OD600 equiv.) for each method under low glucose and glucose-free conditions: (a) Alkaline lysis at RT or on ice, (b) TCA (Waite KA, et al. J Biol Chem. 2016), (c) Glass beads (Li J, et al. J Cell Sci. 2016), and (d) SDS loading buffer lysis with heating to 100˚C (Papa FR, et al. Nature. 1993). In the comparisons, we found that the TCA method could not extract proteins and protein fragments as well, especially under low glucose conditions, when comparing low glucose versus glucose-free conditions (Fig on the right). By contrast, all the other methods extracted similar proteasome subunit fragments. In line with the immunoblot analyses, our EM data showed a large amount of intravacuolar tubules and vesicles that associated with proteasome CPs in *atg8*Δ *pep4*Δ *prb1*Δ mutant cells. This strongly suggests that proteasome fragments are generated during ESCRT-dependent microautophagy. We concluded that alkaline lysis is a better method for protein extraction than TCA, at least under the conditions used in this study.

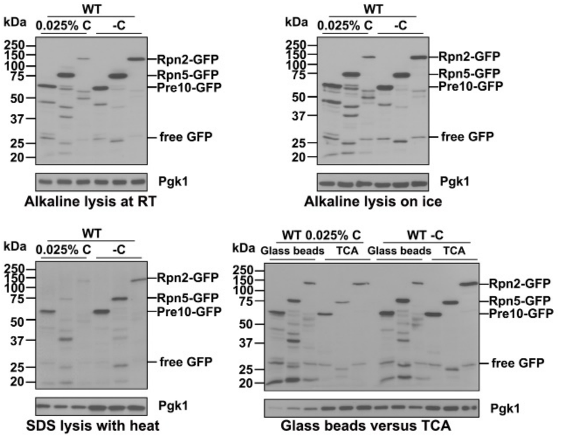

Reviewer #3: In this manuscript, Li et al. explore the mechanism of proteasome degradation during glucose starvation in yeast. In a genome-wide screen for deletion mutants defective in this process they identified AMPK and ESCRT. The effect of these mutations on the dynamics of PSGs is determined here using microscopy and immune-blot analyses of proteasomal proteins. Based on their results the authors draw a number of major decisions. First, under nitrogen starvation ESCRT, but not AMPK, plays a role in macro-autophagy of the proteasome. In contrast, ESCRT and AMPK mediate the assembly under glucose depletion and disassembly when glucose is added back, respectively, of PSGs and their delivery to the lysosome through micro-autophagy. Second, mostly aberrant proteasomes are delivered to the vacuole via micro-autophagy. Third, without AMPK, CT, but not RP, are sequestered into iPOD, showing different regulation of the two parts of proteasomes.
It was previously shown that while macro-autophagy is important during nitrogen starvation, micro-autophagy is important during glucose starvation. It is also known that ESCRT plays a role in both macro- and micro-autophagy. Evidence presented here shows that this is true also for

proteasomes. Because proteasome homeostasis is crucial for cells, this is valuable information. The really novel conclusions are identification of AMPK as a regulator of micro-autophagy and of iPOD as an alternative to PSGs for CP. Showing preferential delivery of aberrant proteasomes for degradation is also important.

We thank the reviewer for the positive comments.

I have a number of criticisms and suggestions that I think would improve the manuscript

Major criticism:

1. Evidence for micro-autophagy is shown in Figure 6. The data needs quantification and a statement of the number of repeats (this is true for a number of other results, especially figure 7). The microscopy and EM analyses need controls of cells grown with glucose and under nitrogen starvation, and most importantly, to show AMPK mutant control. Finally, I suggest using the mutants with aberrant proteasomes (Figure 7) in this experiment.

We thank the reviewer for this suggestion, which is in line with the other two reviewers' suggestions. We have now quantified our EM images and added three figure panels as Fig 6F, 6G, and 6H in the new Fig 6. We also quantified Figs 1-3 and S2 Fig and added the quantification results as Fig 1E in the new Fig 1, Fig 2C in the new Fig 2, and Fig 3C in the new Fig 3, as well as Fig S2C in the new Fig S2 in the supporting information section. We also added a statement of "Each experiment was repeated at least three times" in the materials and methods section. We quantified our EM images in three ways: (1) the percentage of cell sections with the indicated structures in the vacuole: autophagic bodies (ABs), vesicles, ABs & vesicles, and none; (2) the percentage of these structures with gold particle staining; and (3) the percentage of intravacuolar gold particles that associated with the indicated structures in the cells. The text was modified to reference these new quantification data.

We now have added the AMPK mutant control strain *snf4*Δ *atg8*Δ *pep4*Δ *prb1*Δ to the immunogold EM analysis. The prediction was that this would largely eliminate the microautophagy pathway, as was the case for *vps4*Δ *atg8*Δ *pep4*Δ *prb1*Δ, and given the block to macroautophagy (*atg8*Δ), the vacuoles were predicted to be largely empty. Gratifyingly, this is exactly what was observed (Fig 6D and 6F).

We also examined nitrogen-starved *atg8*Δ *pep4*Δ *prb1*Δ cells by immunogold EM. We observed ~50% *atg8*Δ *pep4*Δ *prb1*Δ cell sections had membrane tubules and vesicles in the vacuole and the other ~50% were blank. The appearance of membrane tubules and vesicles in some of the vacuoles in this mutant is anticipated to have resulted from glucose exhaustion during the ~1 day nitrogen starvation. This result is in line with the Vph1-GFP degradation pattern in *atg8*Δ cells under nitrogen starvation (Fig 6A). Lastly, we did not have time to test the proteasome mutants by immuno-EM but agree this is a good experiment for the future.

2. "Proteasome fragmentation" is partial degradation of proteasomal proteins. The authors need to discuss why would the way proteasomes get to the vacuole would determine whether they would be fully or partially degraded. Is it true also for other cargos?

We thank the reviewer for this suggestion. We propose that the microautophagy-associated vesicles and tubules limit proteasome subunit accessibility to the vacuolar proteases. As shown in Fig 6D, proteasomes were mostly within apparent double membranes of the vesicles in the vacuole in *atg8*Δ *pep4*Δ *prb1*Δ cells. We added this speculation to the Discussion.

In terms of partial degradation of other cargos, this appears to be true for the ER membrane protein Sec63-GFP and Rtn1-GFP degradation under ER stress conditions. In a previous study on ER microautophagy, the authors reported three Sec63-GFP fragments larger than free GFP (Fig 4C in Schuck et al. J Cell Sci. 2014).

3. Figure 1: PSGs are identified as fluorescent blobs of a single tagged marker. I think that at least two markers should be used together.

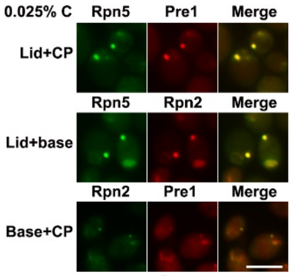

We performed double labeling of proteasome subunits with different combinations between CP, lid, and base subunits. The proteasome subunits we examined colocalized at same PSGs (Fig on the right). The results are consistent with earlier studies (Laporte et al. J Cell Biol. 2008 and Peters et al. J Cell Sci. 2016).

4. Figure 2: The statement in the title (page 8) that ESCRT is required for macro-autophagy is not justified. A comparison between atgXΔ and ESCRT mutants would show whether it is required or plays a role.

We thank the reviewer for this constructive suggestion. In Fig 2 we demonstrated the effects of ESCRT mutants in several ways but had not included the *atg* mutant controls. Here we show immunoblot results of Pre10-GFP, Rpn5-GFP, and Rpn2-GFP in macroautophagy mutants *atg8*Δ, *atg11*Δ, *atg17*Δ, and *atg39*Δ under nitrogen starvation (Fig on the right). In fact, we analyzed macroautophagy mutants and ESCRT mutants at the same time but on separate SDS-PAGE gels, but exposed the membranes to the same film. The results were consistent with previous analysis (Fig 3 in Nemec et al. J Biol Chem. 2017). The main conclusion is that proteasome macroautophagy requires macroautophagy core components (Atg8) and non-selective macroautophagy components (Atg17), but not selective macroautophagy components (Atg11, Atg39) under nitrogen starvation.

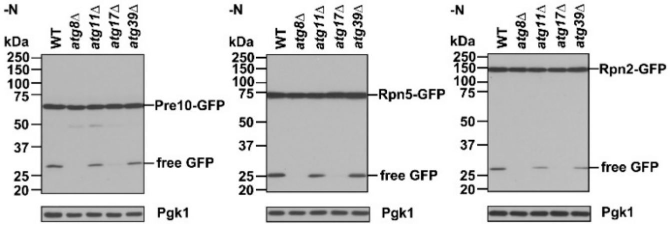

5. Figure 2: How do the authors explain that CP is not delivered in the vacuole in vps4Δ, while it is delivered in other ESCRT mutants?

CP autophagy was mildly inhibited in *vps4*Δ cells while severely inhibited in other ESCRT mutants. CP was able to be delivered in the vacuole in *vps4*Δ cells while not in other ESCRT mutants. We are not sure of why Vps4 is required to a lesser degree, but in the revised text we suggest the idea that the RP base ATPase may somehow compensate for the Vps4 ATPase and help disassemble ESCRT-III complex to promote the delivery of CP with ESCRTs in the vacuole. We make clear this is a speculation.

6. Figure 3 is confusing: A. Under glucose starvation free GFP is seen in all mutants. Why? ESCRT mutants look like WT: why the authors conclude that ESCRT is involved? B. Why PSG co-localize with compartment E?

The free GFP is a signature degradation product of proteasome macroautophagy because free GFP release was blocked in *atg8*Δ cells (Fig 5A). Meanwhile, larger proteasome-GFP fragments were largely inhibited or abolished in ESCRT and AMPK mutant cells. The proteasome fragments are generated by an alternative degradation pathway that requires ESCRTs and AMPK but is independent of core macroautophagy components. Furthermore, we showed cytological evidence for ESCRT-dependent microautophagy of proteasomes that contribute to the complex proteasome fragments (Fig 6D-6H).

PSGs do not colocalize with compartment E in WT cells. A small fraction of proteasome granules were blocked at the perivacuolar region before entering the vacuole and colocalized with Vph1-GFP at class E-like compartments in ESCRT mutants. We also do not fully understand this. The colocalization pattern might indicate a role of ESCRTs in proteasome trafficking to the vacuole, such as through MVBs. Further investigation will be needed to understand the mechanistic roles of ESCRTs in proteasome trafficking.

Other points:

1. It would be good to show that the mutant cells used here are alive after 4-7 days in glucose deprivation.

We thank the reviewer for this valuable suggestion. We now have added the result as S7 Fig in the supporting information section. We performed cell viability assay by PI staining, and analyzed WT, ESCRT mutants *vps27*Δ, *vps28*Δ, *vps25*Δ, *snf7*Δ, *vps4*Δ, AMPK mutants *snf1*Δ and *snf4*Δ, and aberrant proteasome mutants *sem1*Δ and *pre9*Δ after 4-day and 7-day low glucose starvation. We did not perform cell viability assays for the macroautophagy mutants and vacuolar protease-deficient mutants because PSG formation and dissipation were normal in these mutants as compared to WT cells. The results showed that the ESCRT and AMPK mutants were still alive after 4-7 days of glucose starvation at levels comparable to WT cells. AMPK mutants were the least viable mutants (~60-70% viable at day 4 and ~37-65% at day 7), which is expected as the roles of AMPK in general energy homeostasis under glucose starvation conditions.

2. Clarify if PSGs form also under nitrogen starvation

No PSGs are formed under nitrogen starvation as shown in our work (Fig 2B), and also in previous studies, such Marshall et al. Cell Rep. (2016) and Waite et al. J Biol Chem. (2016).

3. Page 18, line 6: "probably facilitating sealing of … autophagosomes": Shown in Zhou et al., JCB 2015.

We cited Zhou et al. J Cell Biol. (2019) in the second part of this sentence (Ref 50) to support our hypothesis; this paper showed that ESCRT factors are directly implicated in sealing autophagosomes.

4. Specify throughout the text and legends whether proteasome subunits used are CP or RP.

We now have specified proteasome subunits used are CP or RP (lid or base) subunit when we first mentioned in the text in lines 146-147, line 182, and lines 371-372, as well as in the figure legends in lines 820-821, line 846, and line 1015.
